# A suppurated, fistulized lymphadenitis in the neck: The untamed tuberculosis

**DOI:** 10.1002/ccr3.3460

**Published:** 2020-10-29

**Authors:** Arben Pilaca, Dhimitër Argjiri, Iris Koshovari, Gentian Vyshka

**Affiliations:** ^1^ Internal Diseases Department International Hospital Tirana Albania; ^2^ Faculty of Medicine University of Medicine in Tirana Tirana Albania

**Keywords:** extrapulmonary tuberculosis, lymphadenitis, neck swelling

## Abstract

Cervical lymphadenopathy is the most frequent form of extrapulmonary tuberculosis. Even in previously endemic countries where tuberculosis is well controlled, tuberculous lymphadenitis needs to be included in the differential diagnosis.

## Graphical abstract

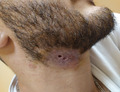

A 35‐year‐old Albanian man presented at our facility in Tirana with a 3‐month history of bilateral, gradual swelling of the front of his neck.

He had emigrated from Albania six and a half years previously and had been itinerant with no fixed address and poor access to medical care. He was diagnosed with dental disease during an emergency visit to a dentist and provided with a 2‐week course of antibiotics, which did not resolve his symptoms. He complained of general malaise, but was able to work. He reported no cough, fever, or weight loss.

On examination, he was found to have a fluctuating palpable mass on the front of his neck, which was more marked on the right and small discharging fistulae (Figure 1A,B). The lymph nodes were fixed, and the overlying skin was indurated. The discharge was whitish and viscous. On chest auscultation, he was found to have crackles and rhonchi at the base of his right lung.

**FIGURE 1:**
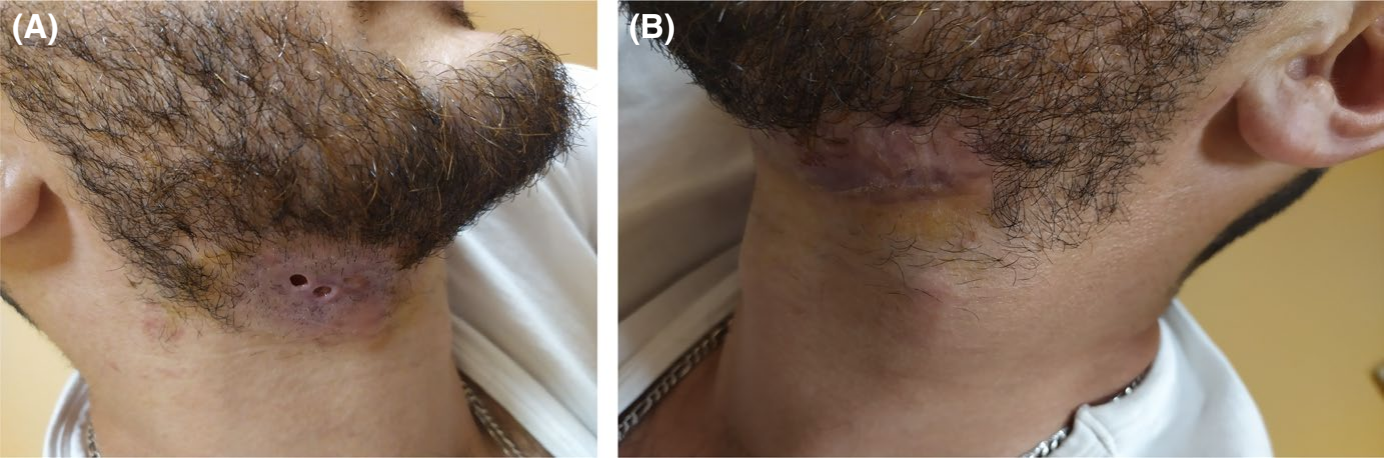
Swollen and hyperemic palpable nodes in the right anterior cervical area, leaking a whitish liquid when slightly pressed

Laboratory tests revealed an elevated erythrocyte sedimentation rate (40 mm/h; reference: 3‐5 mm/h) and an elevated C‐reactive protein level (24 mg/L; reference: < 10 mg/L).

A plain chest radiograph revealed cavitating tuberculosis (Figure 2A,B).

**FIGURE 2:**
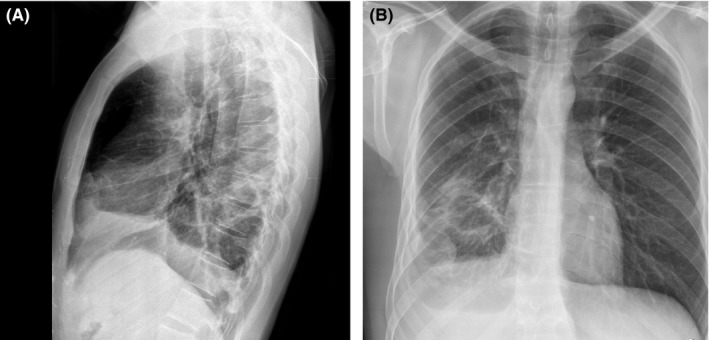
A round opacity in the lower lobes of right lung raised suspicion about a previously not diagnosed, untreated tuberculosis

Sputum and cervical lymph node aspirate were both positive for *Mycobacterium tuberculosis* on Ziehl‐Neelsen stain, and Xpert MTB/RIF (Cepheid Inc) revealed that the strain was nonresistant to rifampicin. The patient was referred to a specialized center for further evaluation, and two samples were tested for drug susceptibility using the Mycobacteria Growth Indicator Tube 960 system (MGIT 960; Becton Dickinson Microbiology Systems).

The patient was started on standard therapy for tuberculosis, with a regimen of four antitubercular drugs (isoniazid, rifampicin, pyrazinamide, and ethambutol).

Diagnosis of tuberculous cervical lymphadenopathy is a challenge.^1^ Cervical lymphadenopathy is the most frequent form of extrapulmonary tuberculosis, but bilateral tuberculous cervical lymphadenopathy is uncommon. Although Albania is not classified as a high‐tuberculosis‐burden country, this condition needs to be considered in the differential diagnosis of cervical lymphadenopathy.^2^

## CONFLICT OF INTEREST

The authors have nothing to disclose.

## AUTHOR CONTRIBUTIONS

AP, DA, and IK: served as the treating clinicians that diagnosed and followed up the case and performed imaging/photographing of the patient. GV: wrote the manuscript and reviewed the literature. All authors: approved the final version.
